# Fistule vésico-vaginale néoplasique secondaire à une tumeur de vessie: une entité rare

**DOI:** 10.11604/pamj.2016.25.59.9969

**Published:** 2016-10-03

**Authors:** Tidiani Bagayogo, Nabil Jakhal, Amine Slaoui, Imad Ziouziou, Tariq Karmouni, Khalid El Khader, Abdelatif Koutani, AhmedI bn Attya Andaloussi

**Affiliations:** 1 Urologie B, Hôpital Ibn Sina Rabat, Maroc

**Keywords:** Fistule vésico-vaginale, tumeurs malignes, cancer de vessie, Vesico-vaginal fistula, malignant tumors, bladder cancer

## Abstract

La fistule vésico-vaginale sur tumeur de vessie est extrêmement rare. Nous rapportons un cas de fistule vésico-vaginale (FVV) néoplasique chez une patiente de 54 ans, ménopausée, sans antécédent chirurgical ou de traumatisme obstétrical récent. La biopsie des berges de la fistule est revenue en faveur d’un carcinome urothélial. A la lumière de cette observation, nous discutons les formes étiologiques ainsi que les différentes stratégies thérapeutiques respectives des FVV.

## Abstract (en)

Vesico-vaginal Fistula (VVF) secondary to bladder cancer is extremely rare. We report the case of a 54-year old post-menopausal patient with neoplasic Vesico-vaginal Fistula (VVF), without any surgical antecedents or recent obstetric trauma. Biopsy of the edges of the fistula confirmed urothelial carcinoma. In light of this observation, we here discuss VVF etiological forms and their different therapeutic strategies.

## Introduction

La fistule vésico-vaginale (FVV) est une communication acquise entre la vessie et le vagin entrainant une fuite permanente d’urine par le vagin. Il s’agit d’une affection très ancienne déjà décrite à l’époque des Pharaons [1]. Elles sont encore très fréquentes dans les pays pauvres où l’accès aux soins des femmes enceintes est restreint, résultant souvent d´accouchements dystociques. Les FVV néoplasiques sont rares, posant souvent un problème à la fois diagnostic et thérapeutique avec les autres fistules. Nous rapportons à travers ce travail un cas d’une FVV secondaire à un carcinome urothélial.

## Patient et observation

Nous présentons le cas clinique d´une patiente âgée de 54ans, sans antécédent notable, avec une parité de 4 enfants, ménopausée depuis 3 ans, qui a rapporté une notion de métrorragies intermittentes depuis 6 mois avec des fuites urinaires permanentes par voie vaginale. L’examen clinique a mis en évidence : patiente apyrétique, en bon état général, conjonctives légèrement décolorées, abdomen souple sans masse palpable et aires ganglionnaires libres. L’examen gynécologique a retrouvé une mauvaise trophicité vaginale, avec visualisation au niveau de la paroi antérieure du vagin un orifice fistuleux à 4 cm du méat avec un col non visible. Au toucher vaginale : le 1/3 inferieur du vagin était souple, les 2/3 supérieurs fibreux. On palpait les berges de la fistule, mais pas le col utérin qui était rétracté vers le haut.

**Figure 1:**
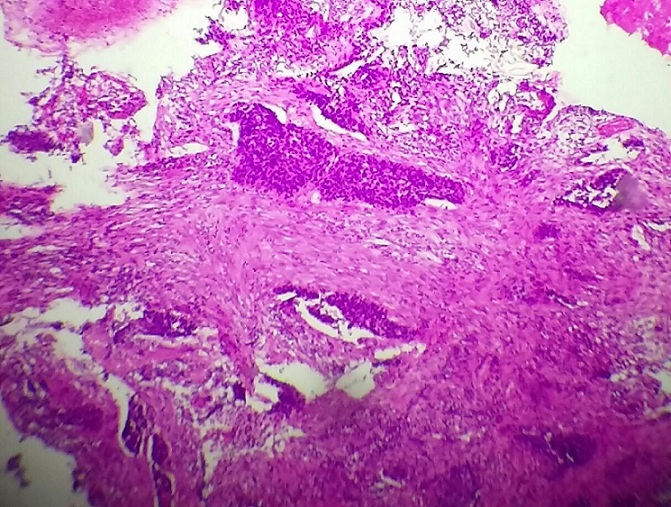
Fragment de biopsie siège de boyaux de cellules tumorales à différenciation urotheliale

**Figure 2:**
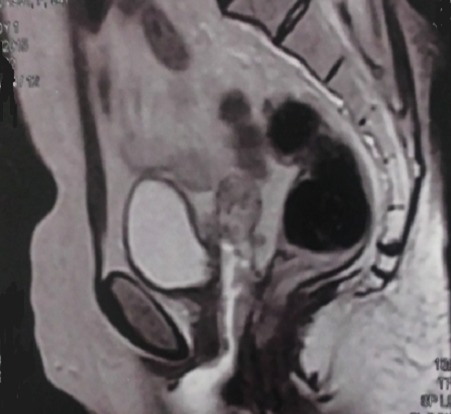
IRM, en séquence T2, montrant une fistule vésico-vaginale avec épaicissement de la paroi vésicale postérieure en regard

Sur le plan biologique, la patiente avait un taux d’hémoglobine à 7 g/dl corrigé par la transfusion et une fonction rénale normale. L’échographie abdomino-pelvienne a objectivé : une urétéro-hydronéphrose droite sans obstacle nettement visible, avec un épaicissement de la paroi vésicale. Une biopsie par voie vaginale de la FVV réalisée est revenue en faveur d’un processus carcinomateux compatible avec une tumeur urothéliale (Figure 1). L’IRM confirme les données antérieures : fistule vesico-vaginale, épaicissement de la paroi postérieure de vessie, urétéro-hydronéphrose droite, sans envahissement ganglionnaire (Figure 2) ou métastatique a distance.

La patiente a subi une pelvectomie antérieure avec dérivation urinaire de type Bricker. L’examen anatomopathologique est revenu en faveur d’un carcinome urothélial de haut grade, infiltrant toute la paroi vésicale, avec marges positives au niveau des recoupes urétrale et vaginale. Les suites opératoires étaient simples et la patiente a été adressée pour une chimiothérapie adjuvante.

## Discussion

Les fistules urogénitales représentent un problème majeur de la santé mondiale, responsable d’importantes morbidités physique, sociale, et psychologique. On estime qu´au moins 3 millions de femmes dans le monde ont une fistule non traitée, tandis qu´entre 30 000 et 130 000 nouvelles fistules sont enregistrées chaque année en Afrique [2].

Le diagnostic différentiel peut se poser avec le syndrome de Youssef (fistule vésico-utérine). Néanmoins, l’urographie intraveineuse (UIV), l’urèthrocystographie rétrograde et mictionnelle (UCRM), l’uro-scanner ou même l’uro-IRM peuvent permettent de trancher [3].

Selon Benchekroun les fistules peuvent être classées en fonction du site: type I fistule urétro-vaginale (30 %); type II fistule cervico-vaginale (22 %); type III fistule vésico-vaginale (48 %) [4].

Les FVV peuvent être provoquées par plusieurs causes : iatrogène (une intervention chirurgicale), un traumatisme, une infection, une maladie inflammatoire de l’intestin, et les tumeurs malignes gynécologiques ou d´autres organes pelviens [5].

Les FVV post-obstétricales sont les plus fréquentes dans les pays pauvres, estimées à 95.2% [6]. Elles sont secondaires à une compression ischémique de la vessie, de l’urètre et du périnée et/ou au traumatisme des forceps [7]. Il s’ensuit des délabrements pelvipérinéaux étendus de mauvais pronostic.

Dans les pays riches, les FVV postopératoires représentent 83.2% [7]. Elles apparaissent à la suite d’une chirurgie pelvienne par voie haute ou basse: hystérectomie, césarienne, cure de prolapsus. Elles sont la conséquence d’une plaie vésicale méconnue en per-opératoire ou une dissection poussée dévascularisant la paroi vésicale [7]. Il s’agit d’une lésion directe, limitée et de bon pronostic. Les techniques de réparation proposées sont nombreuses, du fait de la très grande variété des lésions rencontrées, qui appelle des solutions diverses. Le but recherché par l’opérateur est double : rétablir à la fois la continence des réservoirs, vésical, à savoir une fonction mictionnelle normale ; restaurer une vie conjugale normale avec le retour des menstrues et la possibilité de rapports sexuels et donc de maternité. Cet objectif doit être poussé à l’extrême même au prix d’interventions multiples.

Chez les patientes atteintes d´une tumeur maligne, les fistules surviennent à un stade avancé de la tumeur ou à la suite de son traitement chirurgical ou de son irradiation [6]. Les fistules post-radiques peuvent se manifester plusieurs mois ou années plus tard, et elles pourraient se produire en raison d’une inflammation chronique des petits vaisseaux conduisant à l´ischémie tissulaire [7]. Les FVV néoplasiques, primaires par envahissement tumoral, sont rares et secondaires aux tumeurs gynécologiques: col, ovaires et endomètre [6]. Ainsi le traitement repose sur l’exérèse totale du processus tumoral emportant la fistule. Nous avons classé la tumeur de vessie en stade IVa, N0, M0 avec réalisation d’une pelvectomie antérieure avec dérivation urinaire type Bricker et chimiothérapie adjuvante. Les suites opératoires étaient simples. Et à ce jour la patiente ne rapporte pas de récidive.

## Conclusion

Les FVV obstétricales et postopératoires sont les formes étiologiques les plus fréquentes. Par ailleurs les formes malignes sont rares et surtout celles secondaires aux tumeurs malignes de la vessie.
